# Changing geographical patterns and trends in cancer incidence in children and adolescents in Europe, 1991–2010 (Automated Childhood Cancer Information System): a population-based study

**DOI:** 10.1016/S1470-2045(18)30423-6

**Published:** 2018-09

**Authors:** Eva Steliarova-Foucher, Miranda M Fidler, Murielle Colombet, Brigitte Lacour, Peter Kaatsch, Marion Piñeros, Isabelle Soerjomataram, Freddie Bray, Jan Willem Coebergh, Rafael Peris-Bonet, Charles A Stiller, ACCIS contributors, Monika Hackl, Anna Zborovskaya, Nadya Dimitrova, Zdravka Valerianova, Ladislav Dušek, Margit Mägi, Alain Monnereau, Jacqueline Clavel, Michel Velten, Anne-Valérie Guizard, Véronique Bouvier, Xavier Troussard, Anne-Sophie Woronoff, Emilie Marrer, Brigitte Trétarre, Marc Colonna, Olivier Ganry, Pascale Grosclaude, Berndt Holleczek, Zsuzsanna Jakab, Laufey Tryggvadóttir, Lucia Mangone, Franco Merletti, Stefano Ferretti, Bianca Caruso, Maria Michiara, Rosario Tumino, Fabio Falcini, Roberto Zanetti, Giovanna Tagliabue, Otto Visser, Giske Ursin, Ryszard Mężyk, Kamila Kepska, José Laranja Pontes, Maja Primic Žakelj, Rafael Fernández-Delgado, Marisa L Vicente Raneda, Enrique Almar Marqués, José Ramón Quirós Garcia, Arantza Lopez de Munain, Rafael Marcos-Gragera, Maria-Jose Sanchez-Perez, Maria Ramos Monserrat, Eva Ardanaz, Jaume Galceran, Staffan Khan, Claudia E Kuehni, Christine Bouchardy, Fabio Levi, Isabelle Konzelmann, Sabine Rohrmann, Sally Vernon, David H Brewster, Ceri White, Anastasia Dolya

**Affiliations:** a Section of Cancer Surveillance, International Agency for Research on Cancer, World Health Organization, Lyon, France; b French National Registry of Childhood Solid Tumours, Centre Hospitalier Régional Universitaire, Nancy, France; c Inserm U1153, Epidemiology and Biostatistics Sorbonne Paris Cité Centre (CRESS), Epidemiology of Childhood and Adolescent Cancers Team (EPICEA), Paris, France; d German Childhood Cancer Registry, University Medical Center, Mainz, Germany; e Department of Public Health, Erasmus MC, Rotterdam, Netherlands; f Spanish Registry of Childhood Tumours (RETI-SEHOP), Faculty of Medicine, University of Valencia, Valencia, Spain; g National Cancer Registration and Analysis Service, Public Health England, London, UK

## Abstract

**Background:**

A deceleration in the increase in cancer incidence in children and adolescents has been reported in several national and regional studies in Europe. Based on a large database representing 1·3 billion person-years over the period 1991–2010, we provide a consolidated report on cancer incidence trends at ages 0–19 years.

**Methods:**

We invited all population-based cancer registries operating in European countries to participate in this population-based registry study. We requested a listing of individual records of cancer cases, including sex, age, date of birth, date of cancer diagnosis, tumour sequence number, primary site, morphology, behaviour, and the most valid basis of diagnosis. We also requested population counts in each calendar year by sex and age for the registration area, from official national sources, and specific information about the covered area and registration practices. An eligible registry could become a contributor if it provided quality data for all complete calendar years in the period 1991–2010. Incidence rates and the average annual percentage change with 95% CIs were reported for all cancers and major diagnostic groups, by region and overall, separately for children (age 0–14 years) and adolescents (age 15–19 years). We examined and quantified the stability of the trends with joinpoint analyses.

**Findings:**

For the years 1991–2010, 53 registries in 19 countries contributed a total of 180 335 unique cases. We excluded 15 162 (8·4%) of 180 335 cases due to differing practices of registration, and considered the quality indicators for the 165 173 cases included to be satisfactory. The average annual age-standardised incidence was 137·5 (95% CI 136·7–138·3) per million person-years and incidence increased significantly by 0·54% (0·44–0·65) per year in children (age 0–14 years) with no change in trend. In adolescents, the combined European incidence was 176·2 (174·4–178·0) per million person-years based on all 35 138 eligible cases and increased significantly by 0·96% (0·73–1·19) per year, although recent changes in rates among adolescents suggest a deceleration in this increasing trend. We observed temporal variations in trends by age group, geographical region, and diagnostic group. The combined age-standardised incidence of leukaemia based on 48 458 cases in children was 46·9 (46·5–47·3) per million person-years and increased significantly by 0·66% (0·48–0·84) per year. The average overall incidence of leukaemia in adolescents was 23·6 (22·9–24·3) per million person-years, based on 4702 cases, and the average annual change was 0·93% (0·49–1·37). We also observed increasing incidence of lymphoma in adolescents (average annual change 1·04% [0·65–1·44], malignant CNS tumours in children (average annual change 0·49% [0·20–0·77]), and other tumours in both children (average annual change 0·56 [0·40–0·72]) and adolescents (average annual change 1·17 [0·82–1·53]).

**Interpretation:**

Improvements in the diagnosis and registration of cancers over time could partly explain the observed increase in incidence, although some changes in underlying putative risk factors cannot be excluded. Cancer incidence trends in this young population require continued monitoring at an international level.

**Funding:**

Federal Ministry of Health of the Federal German Government, the European Union's Seventh Framework Programme, and International Agency for Research on Cancer.

## Introduction

Cancer is an important disease burden in children as it is not easily prevented, with known causes explaining only a small proportion of cases. In Europe, incidence rates range from 140–170 per million person-years (ie, number of incident cases divided by the number of person-years at risk) in populations younger than 15 years and from 180–240 per million in those aged 15–19 years.^1^ During the past three decades, incidence increased by about 1% per annum for all cancers combined and this increase affected most major diagnostic groups, including leukaemias, lymphomas, and CNS tumours.^2^ However, in the past decade, incidence appears to have stabilised overall and for the major diagnostic groups in European populations.^3^, ^4^, ^5^, ^6^, ^7^, ^8^, ^9^, ^10^

**Research in context**

**Evidence before this study**

We searched PubMed with no language restrictions to identify studies published since Jan 1, 2004, that investigated cancer incidence trends in children (age 0–14 years) and adolescents (age 15–19 years) in Europe and other high-income countries, using the search terms ‘child* cancer incidence trends’ and ‘adolescen* cancer incidence trends’. Evidence from retrieved studies suggested that after a substantial increase in childhood cancer incidence towards the end of the last century, the rate of increase has declined in the 2000s. However, the limitations of most of these studies, in terms of number of cases or timeframe, might have hampered the full understanding of the dynamisms behind the observed trends in this population.

**Added value of this study**

We used all quality data available in Europe for the full calendar years in the period 1991–2010 to evaluate incidence patterns and trends in children and adolescents. Overall, incidence continued to increase during the study period by 0·5% per year in children and 1·0% per year in adolescents. However, we observed some evidence of a deceleration in increasing cancer trends, modulated by age group, European region, and diagnostic group. Based on the large scale of all available European data, our findings add an authoritative account on the geographical patterns and temporal trends of cancer in children and adolescents in Europe.

**Implications of all the available evidence**

The increasing trends might have resulted from improvements in cancer diagnosis or registration in this age group, although the influence of other factors cannot be excluded. The paucity of evidence of stabilisation of cancer incidence in Europe and the variability of the observed trends support the need for continued international monitoring and research into causal mechanisms.

Leukaemias, lymphomas, and tumours of the CNS represent 70% of all cancers observed in European populations younger than 15 years and half of all cancers in those aged 15–19 years^1^ and therefore contribute considerably to the overall incidence. Changing incidence could result from numerous factors, including variations in diagnostic capacity, completeness of registration, population composition, and exposure to risk factors. Monitoring these trends is important for planning health-care delivery and for aetiological research.

In this study, we documented and interpreted incidence trends in Europe for cancers diagnosed at ages 0–19 years using quality-assured population-based cancer registries. We focused on the 20-year period 1991–2010, assessing the trends separately in children (aged 0–14 years) and adolescents (aged 15–19 years), for all cancers combined and for the major diagnostic groups. Our results update the Automated Childhood Cancer Information System (ACCIS) studies, extending the previously reported period of observation by over a decade.^2^, ^11^

## Methods

### Data acquisition

We invited all population-based cancer registries operating in European countries (as defined by the UN Statistics Division^12^) and Cyprus to participate in this population-based registry study. We requested a listing of individual records of cancer cases, and the population in each calendar year by sex and age from official national sources, accompanied by specific information about the geographical and administrative area covered and registration practices.

Information on cancer cases included coded data on sex, age, date of birth, date of diagnosis, tumour sequence number, primary site, morphology, behaviour, and the most valid basis of diagnosis. Most registries coded tumours according to the International Classification of Diseases for Oncology, Third Edition (ICD-O-3^13^) as required, and International Classification of Diseases for Oncology, Second Edition^14^ codes were converted to ICD-O-3 codes. Subsequently, neoplasms were classified according to the International Classification of Childhood Cancer, Third Edition (ICCC-3).^15^ We examined individual records for internal consistency,^16^ verifying unlikely combinations of site with morphology, age or sex with tumour type, basis of diagnosis with morphology, and rare tumour entities. The proportions of cases microscopically verified, identified from death certificate only, or with unknown basis of diagnosis and unspecified morphology contributed to data evaluation. The ACCIS Scientific Committee assessed the distribution of new cases across years and plausibility of case mix and age distribution. Standardised tables, charts, statistics, lists of selected questioned records, and any relevant information provided by the registry or known from published sources were discussed at meetings of the ACCIS Scientific Committee, who decided on the inclusion of each dataset in the ACCIS database. Only datasets with high-quality data were eligible for inclusion in the analyses.

The cancers included in the analyses were all malignant tumours diagnosed during the complete calendar years 1991–2010, in people younger than 20 years and resident in the contributing registration areas. Several cancer types were excluded because they were not eligible for registration in all participating registries or during the whole study period: myelodysplasias (ICD-O-3 M-codes starting with 998), pilocytic astrocytoma (ICD-O-3 code M-9421), non-melanoma skin cancer (ICCC-3 subgroup XIe and XIIb with site code C44), and carcinoid tumour of the appendix (ICD-O-3 site code C18.1 and M-8240).

The data used in this study were submitted and validated during the years 2015–16. Our study design was reviewed and approved by the International Agency for Research on Cancer (IARC) Ethics Committee on June 17, 2015.

### Dataset constitution

An eligible registry could become a contributor if it provided quality data for all complete calendar years in the 20-year period 1991–2010. The 53 contributing registries in 19 countries (appendix p 2) included nine paediatric registries. The paediatric registries collected and provided data for those aged 0–14 years, and the other cancer registries and the paediatric registry of Belarus provided data for the full target age range (0–19 years). The French national paediatric registry registered haematological malignancies only.

Some populations in France, Germany, Italy, Spain, Switzerland, and the UK were covered by both paediatric and general cancer registries. To avoid double counting of cases in two registries while using the maximum number of cases for analyses in these populations, we allocated each registry to one or more datasets (appendix p 2). We built three datasets—one with 39 registries contributing to the analyses of all cancers, CNS tumours, and other tumours in ages 0–14 years, a second with 32 registries contributing to the analyses of leukaemia and lymphoma in ages 0–14 years, and a third with 45 registries contributing to all the analyses of those aged 15–19 years. The first and second datasets (ages 0–14 years) differed only in the contribution from France; the analyses of all cancers and CNS tumours used a dataset that included data from the French general cancer registries, while the leukaemia and lymphoma dataset included data from the French national paediatric registry of haematological malignancies. The French national registry increased the person-years by ten times and provided 11 770 more haematological malignancies compared with the combined contribution of the French regional registries.

Subnational numbers of cases and person-years were pooled to produce national cancer incidence and countries were further pooled into four European regions (appendix p 2) according to UN definitions.^12^ The person-years available in each region are shown in the appendix (p 3).

### Statistical analysis

The number of incident cases in the covered areas of the participating registries during the study period determined our sample size. Incidence was calculated as the number of cases divided by the number of person-years in the categories of geographical area, sex, age, and diagnostic group for the given 20-year period and expressed per million person-years. As the age distribution of population at risk differs between countries and over time, we adjusted the reported incidence for the age range 0–14 years for age via direct standardisation using weights 12, 10, and 9 for the three age groups 0–4 years, 5–9 years, and 10–14 years, respectively.^17^ We also calculated 95% CIs. As each combination of calendar year, sex, and age group had a positive person-years count, we encountered no missing data.

To graphically portray incidence trends for all cancers, we plotted observed incidence against calendar year for each country, and the rates for the four European regions were smoothed using a locally weighted regression^18^ of the incidence on year.

To assess the average annual percentage change, we fit the natural logarithm of the incidence with year using generalised linear regression models adjusting for age group and region, as appropriate. We quantified changes in incidence as the average annual percentage change, with corresponding 95% CIs. The null hypothesis corresponded to no change in the annual rate, which was equivalent to 0 lying within the 95% CI for the average annual percentage change. We analysed each cancer category, overall and by European region, using Stata (version 14). We examined incidence trends for changes during the study period using Joinpoint Regression Program (version 4.1.0) applied to the log rates, separately for the age groups 0–14 years and 15–19 years in the total dataset, and in each cancer category, overall and by region. The null hypothesis assumed the annual percentage change was constant throughout the study period. We used the permutation test^19^ to determine the number of joinpoints, by default set to a maximum of three for Europe and five for analyses adjusted for region. Where joinpoints were detected, we reported the annual percentage change with corresponding 95% CIs for each of the linear segments identified between two significant joinpoints.

### Role of the funding source

The funders of the study external to the collaborating institutions had no role in study design, data collection, data analysis, data interpretation, or writing of the report. MC, MMF, and ES-F had full access to all the data used in the study. The corresponding author had final responsibility for the decision to submit for publication.

## Results

During the full calendar years of the period 1991–2010, the 53 registries contributed 1·3 billion person-years and 180 335 unique cases. 15 162 (8·4%) of 180 335 cases were excluded, and the proportion of excluded cases varied slightly between age groups and regions. We considered the quality indicators for the 165 173 included cases to be satisfactory (appendix p 3). In 2010, the available population at risk represented 53·2 million (46·6%) of 114·1 million of the European population aged 0–14 years and 9·8 million (22·6%) of 43·2 million aged 15–19 years, with reference to UN population estimates.^20^ In western Europe, coverage of the childhood population was almost complete for haematological malignancies (27·9 million [93·3%] of 29·9 million), and 17·5 million (58·5%) of 29·9 million were covered for the other malignancies, while 2·1 million (20·1%) of 10·6 million adolescents were covered (appendix p 3).

**Figure 1:**
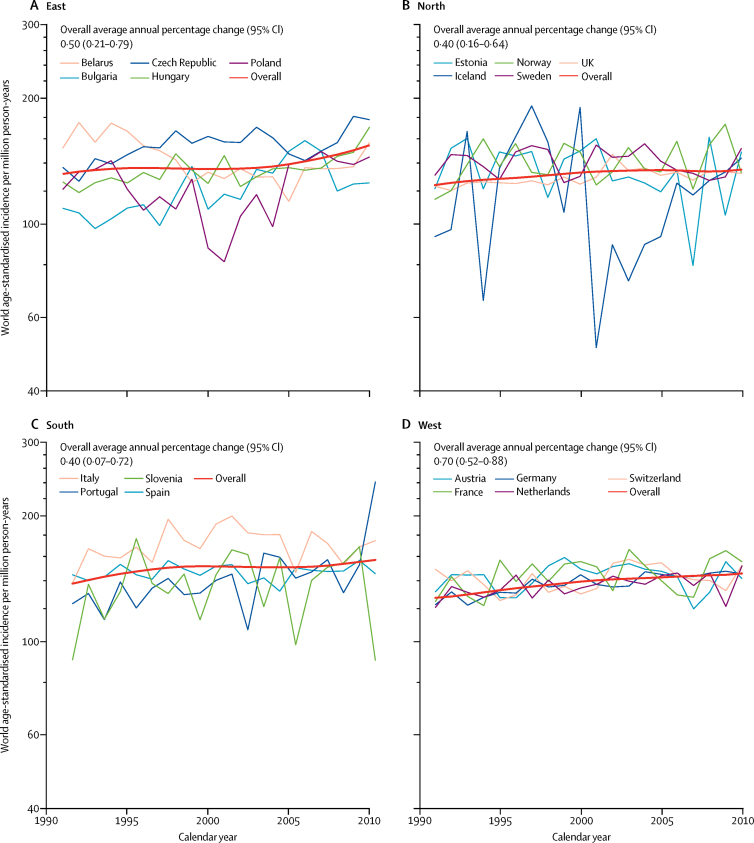
Incidence trends of cancer in children aged 0–14 years in Europe, 1991–2010 Jagged thin lines indicate annual age-standardised rates in countries and smooth red thick lines indicate modelled incidence trends in regions.

**Figure 2:**
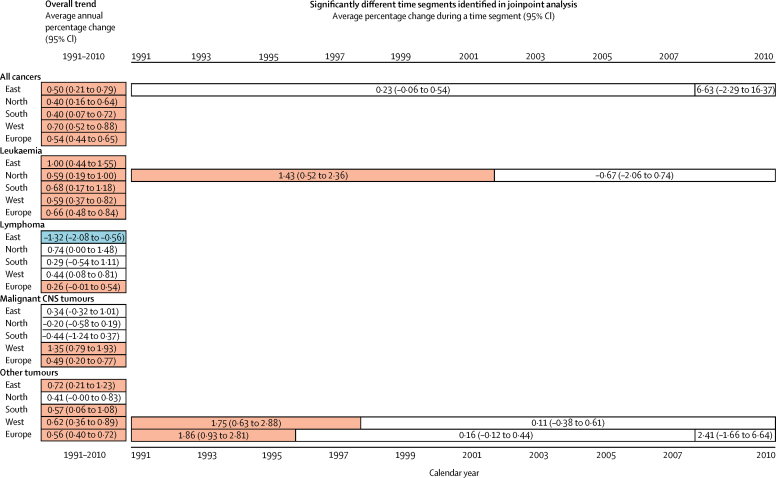
Overview of observed incidence trends of cancer in children aged 0–14 years for the entire study period and for any time segments identified in joinpoint analysis, by diagnostic group and region of Europe, 1991–2010 Red indicates an increasing trend, blue indicates a decreasing trend, and white indicates no significant trend. Absence of bars indicates no joinpoint was identified for a given category.

We included all 118 265 eligible cases in patients aged 0–14 years in our analyses of incidence of all cancers. The average annual age-standardised incidence was 137·5 (95% CI 136·7–138·3) per million person-years and incidence increased significantly by 0·54% (0·44–0·65) per year on average (table 1), with no break in the time trend. There was a large decrease in incidence for the national registry of Belarus, contributing to the regional pattern (figure 1). The large fluctuation of annual incidence in Iceland was due to small population size and did not visibly affect the shape of the regional curve (figure 1). The trend in the east was split into two segments, both with non-significant trends (figure 2).

**Table 1:** Number of new cases, world age-standardised incidence per million person-years, and average annual percentage change by diagnostic category in children aged 0–14 years, by European region, 1991–2010

	**All cancers**			**Leukaemia**			**Lymphoma**			**Malignant CNS tumours**			**Other cancers**		
	Cases	Incidence per million person-years (95% CI)	Annual change (95% CI)	Cases	Incidence per million person-years (95% CI)	Annual change (95% CI)	Cases	Incidence per million person-years (95% CI)	Annual change (95% CI)	Cases	Incidence per million person-years (95% CI)	Annual change (95% CI)	Cases	Incidence per million person-years (95% CI)	Annual change (95% CI)
East	19 274	137·9 (135·9 to 139·9)	0·50% (0·21 to 0·79)	5721	42·6 (41·5 to 43·8)	1·00% (0·44 to 1·55)	2652	17·1 (16·5 to 17·8)	−1·32% (−2·08 to −0·56)	3367	23·6 (22·8 to 24·4)	0·34% (−0·32 to 1·01)	7534	54·5 (53·3 to 55·8)	0·72% (0·21 to 1·23)
North	34 339	131·5 (130·1 to 132·9)	0·40% (0·16 to 0·64)	12 015	47·0 (46·1 to 47·8)	0·59% (0·19 to 1·00)	3848	13·5 (13·0 to 13·9)	0·74% (0·00 to 1·48)	5996	22·7 (22·1 to 23·2)	−0·20% (−0·58 to 0·19)	12 480	48·4 (47·6 to 49·3)	0·41% (−0·00 to 0·83)
South	14 450	149·9 (147·4 to 152·3)	0·40% (0·07 to 0·72)	4472	47·3 (45·9 to 48·7)	0·68% (0·17 to 1·18)	2137	20·4 (19·5 to 21·2)	0·29% (−0·54 to 1·11)	2317	23·9 (22·9 to 24·8)	−0·44% (−1·24 to 0·37)	5524	58·3 (56·8 to 59·9)	0·57% (0·06 to 1·08)
West	50 202	138·2 (136·9 to 139·4)	0·70% (0·52 to 0·88)	26 250	47·8 (47·2 to 48·3)	0·59% (0·37 to 0·82)	9821	15·8 (15·5 to 16·1)	0·44% (0·08 to 0·81)	7733	21·0 (20·5 to 21·4)	1·35% (0·79 to 1·93)	18 168	51·2 (50·5 to 52·0)	0·62% (0·36 to 0·89)
Europe	118 265	137·5 (136·7 to 138·3)	0·54% (0·44 to 0·65)	48 458	46·9 (46·5 to 47·3)	0·66% (0·48 to 0·84)	18 458	15·8 (15·6 to 16·0)	0·26% (−0·01 to 0·54)	19 413	22·2 (21·9 to 22·6)	0·49% (0·20 to 0·77)	43 706	51·7 (51·2 to 52·2)	0·56% (0·40 to 0·72)

We investigated incidence for four diagnostic groups in patients aged 0–14 years (table 1, figure 2, appendix p 4), and included all eligible cases. The combined age-standardised incidence of leukaemia based on 48 458 cases in patients aged 0–14 years was 46·9 (95% CI 46·5–47·3) per million person-years and increased by 0·66% (0·48–0·84) per year, with no change in slope. We observed an increase in each region, although only during the first decade in the north. The overall age-standardised incidence of lymphoma was 15·8 (15·6–16·0) per million person-years (based on 18 458 cases) and varied modestly by region. Incidence increased in the west and decreased in the east, and we detected no change in the slopes. The overall age-standardised incidence of malignant CNS tumours (19 413 cases) of 22·2 (95% CI 21·9–22·6) per million person-years was observed to rise at 0·49% (0·20–0·77) per annum with no joinpoints; by region, we observed an increasing trend only in the west, as the rates were stable in the three other regions.

In patients aged 0–14 years, the combined incidence of all 43 706 other cancers increased in Europe overall and in all regions except the north. In joinpoint analysis, the European trend increased only over the first few years, similar to the trend in the west (figure 2).

**Figure 3:**
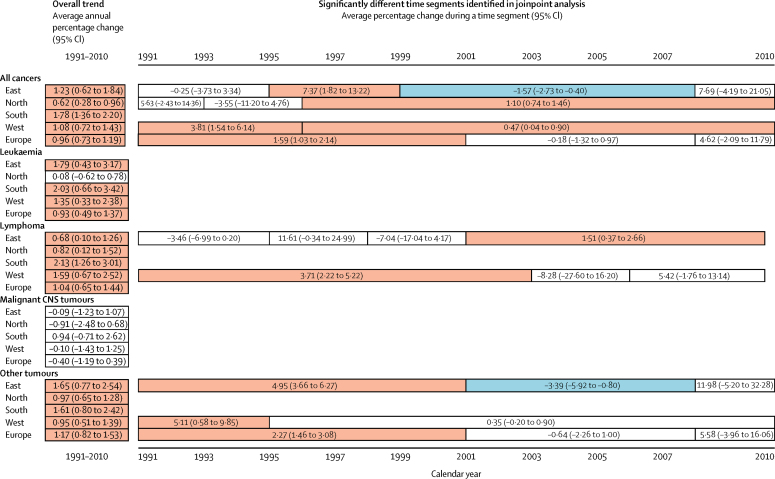
Overview of observed incidence trends of cancer in adolescents aged 15–19 years for the entire study period and for any significant time segments identified in joinpoint analysis, by diagnostic group and region of Europe, 1991–2010 Red indicates an increasing trend, blue indicates a decreasing trend, and white indicates no significant trend. Absence of bars indicates no joinpoint was identified for a given category.

**Figure 4:**
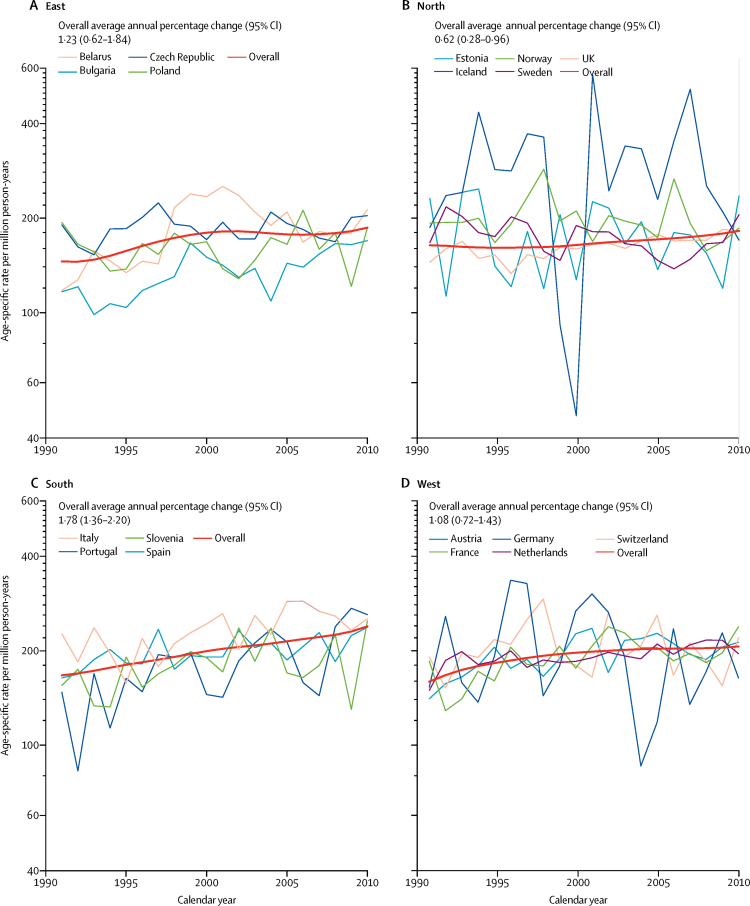
Incidence trends of cancer in adolescents aged 15–19 years in Europe, 1991–2010 Jagged thin lines indicate annual age-specific rates in countries and smooth thick red lines indicate modelled incidence trends in regions.

In adolescents (aged 15–19 years), the combined European incidence was 176·2 (95% CI 174·4–178·0) per million person-years based on all 35 138 eligible cases and incidence increased by 0·96% (95% CI 0·73–1·19) per year in the period 1991–2010 (table 2), although no significant change in trend was seen in the second decade (figure 3). We investigated incidence trends by country and region, and regional trends by diagnostic group (figure 4, appendix p 5). In addition to large variations due to small numbers of cases for some countries, we observed a large range in incidence (between around 140 and 240 per million person-years) for Belarus. The slopes of trends in the regions were uneven, except in the south. In the east, a decrease was noted over the time segment 1999–2008 (figure 3).

**Table 2:** Number of new cases, age-specific incidence per million person-years, and average annual percentage change by diagnostic category in adolescents aged 15–19 years, by European region, 1991–2010

	**All cancers**			**Leukaemia**			**Lymphoma**			**Malignant CNS tumours**			**Other cancers**		
	Cases	Incidence per million person-years (95% CI)	Annual change (95% CI)	Cases	Incidence per million person-years (95% CI)	Annual change (95% CI)	Cases	Incidence per million person-years	Annual change (95% CI)	Cases	Incidence per million person-years (95% CI)	Annual change (95% CI)	Cases	Incidence per million person-years (95% CI)	Annual change (95% CI)
East	8041	169·6 (165·9 to 173·4)	1·23% (0·62 to 1·84)	1023	21·6 (20·3 to 22·9)	1·79% (0·43 to 3·17)	2096	44·2 (42·3 to 46·1)	0·68% (0·10 to 1·26)	749	15·8 (14·7 to 16·9)	−0·09% (−1·23 to 1·07)	4173	88·0 (85·4 to 90·7)	1·65% (0·77 to 2·54)
North	15 207	167·2 (164·5 to 169·8)	0·62% (0·28 to 0·96)	2192	24·1 (23·1 to 25·1)	0·08% (−0·62 to 0·78)	3981	43·8 (42·4 to 45·1)	0·82% (0·12 to 1·52)	1358	14·9 (14·1 to 15·7)	−0·91% (−2·48 to 0·68)	7676	84·4 (82·5 to 86·3)	0·97% (0·65 to 1·28)
South	3941	197·0 (190·8 to 203·1)	1·78% (1·36 to 2·20)	501	25·0 (22·8 to 27·2)	2·03% (0·66 to 3·42)	1244	62·2 (58·7 to 65·6)	2·13% (1·26 to 3·01)	288	14·4 (12·7 to 16·1)	0·94% (−0·71 to 2·62)	1908	95·4 (91·1 to 99·6)	1·61% (0·80 to 2·42)
West	7949	193·7 (189·4 to 197·9)	1·08% (0·72 to 1·43)	986	24·0 (22·5 to 25·5)	1·35% (0·33 to 2·38)	2125	51·8 (49·6 to 54·0)	1·59% (0·67 to 2·52)	588	14·3 (13·2 to 15·5)	−0·10% (−1·43 to 1·25)	4250	103·5 (100·4 to 106·6)	0·95% (0·51 to 1·39)
Europe	35 138	176·2 (174·4 to 178·0)	0·96% (0·73 to 1·19)	4702	23·6 (22·9 to 24·3)	0·93% (0·49 to 1·37)	9446	47·4 (46·4 to 48·3)	1·04% (0·65 to 1·44)	2983	15·0 (14·4 to 15·5)	−0·40% (−1·19 to 0·39)	18 007	90·3 (89·0 to 91·6)	1·17% (0·82 to 1·53)

For adolescents, the overall incidence of leukaemia was 23·6 (95% CI 22·9–24·3) per million person-years (based on all 4702 eligible cases) and varied little between regions (table 2). Incidence increased except in the north, where it was fairly stable, resulting in an average annual change of 0·93% (0·49–1·37). No change in the slopes was detected. Lymphoma (9446 cases) occurred more frequently than did leukaemia (4702 cases) or malignant CNS tumours (2983 cases) in adolescents compared with children (table 2), and the overall incidence of 47·4 (46·4–48·3) per million person-years varied moderately by region. An increase in incidence was observed for all European data combined and in all European regions investigated. Changes in this trend were detected in the east and west, where the increase was limited to the second and first half of the study period, respectively (figure 3). The overall age-specific incidence of malignant CNS tumours was 15·0 (14·4–15·5) per million person-years. Incidence was stable overall and in the four defined regions (figure 3), with no change in the slopes.

The overall rate for all other cancers in adolescents (18 007 cases) was 90·3 per million. The European avearge annual change of 1·17% (0·82–1·53) differed between three segments—among them a significant increase was detected during the first decade only. The pattern of changes in the slopes by region was similar to that observed for all cancers (figure 3).

## Discussion

In this population-based registry study, we reported on cancer incidence trends in the European population of children (aged 0–14 years) and adolescents (aged 15–19 years) during the full calendar years 1991–2010, thus extending the ACCIS study by 10–13^2^, ^11^ years of observation. The overall incidence for children and adolescents increased significantly over this 20-year period.

The increase in incidence was not constant by cancer type, region, or over time, and there was a suggestion of stabilisation in the trend for all cancers in adolescents in the dataset for the whole of Europe (appendix p 6). The most pronounced inter-regional diversities included the decreasing incidence of childhood lymphomas in the east (compared with stable or increasing incidence in the other regions), an increase in the incidence of childhood CNS tumours in the west (compared with little change observed in the other regions), and the stable incidence of leukaemia in adolescents in the north (relative to increasing incidence in the other regions).

Although our results are largely confirmatory and incremental from previous findings, a major strength of our study is its large size, which permits detection of a moderate rate of increase of 0·5% per year in the age group 0–14 years. Data from large paediatric cancer registries helped to increase the coverage and creation of a specific dataset for childhood haematological malignancies enlarged the relevant person-years in the west (583·8 million) by 53% compared with data available for other neoplasms (380·5 million). We examined the trends separately in children and adolescents to allow for variations in trends between these populations with a different case mix.

We validated the quality and completeness of the data from the included registries during a thorough dataset assessment. We consider our results to provide the best available estimates of incidence trends in the European population of children and adolescents for 1991–2010.

A limitation of our study is the variable coverage of the regions, which might affect the representativeness of the incidence at the national and European regional levels. The available data do not allow speculation on what the incidence would be if coverage was complete—increase of coverage and quality of registration is required for a full picture of the cancer burden. Meanwhile, by use of all quality information available for the study period, our results maximise the number of person-years of comparable data, and provide evidence of changing trends in European populations.

We excluded several cancer types that are usually included in reports of childhood cancer incidence from our analyses. The excluded cancer types are classified by the ICCC-3^14^ and contribute to the total cancer burden, and it is therefore preferable that they are registered. In particular, exclusion of non-malignant CNS tumours might have shaped the observed trends. We plan to examine this assumption in a focused analysis of trends of all CNS tumours, by behaviour and diagnostic subgroup, and by considering the differences and changes in coding and their reportability.

Our reported total incidence was 5% to 15% lower (depending on the region) than in another report assessing the period 2001–10.^1^ This disparity is explained by the exclusion of several groups of neoplasms from our study and the period starting one decade earlier, which would, in the presence of an increasing trend, result in a lower overall rate.

The overall average annual change of 0·54% in children aged 0–14 years is lower than the 1% reported by an ACCIS study based on diagnoses in the 1970s through to the 1990s^2^ or for the period 1978–97,^21^ and might indicate a deceleration in the increase of cancer incidence, although the registry datasets included differ somewhat between the studies. A lower rate of increase could suggest the end of an improvement in reporting, but might also reflect reduced reporting discipline, a change in classification of cancers, or other factors. Cancer-specific studies might provide more precise interpretation of these trends.

Studies in European populations of smaller sizes reported stabilising of cancer incidence in children overall and in the three major diagnostic groups.^3^, ^4^, ^5^, ^6^, ^7^, ^8^, ^9^, ^10^ By contrast, data collected in the longstanding registries of the Surveillance, Epidemiology, and End Results (SEER) Program suggest continued minor increases in overall childhood cancer incidence over the most recently assessed period 1995–2014,^22^ consistent with our study. In adolescents aged 15–19 years, varying incidence trends were observed in different European populations for leukaemia, lymphoma, or CNS tumours.^3^, ^4^, ^7^ As for the SEER data,^22^ our study found an increase in overall cancer incidence and leukaemia and lymphoma incidence, but no change in trends for malignant CNS tumours. A large US study^23^ reported stable incidence over the period 2001–09 overall and for the three major diagnostic groups in the population younger than 20 years. We identified changes in overall trends that provide some evidence of a deceleration of time trends during the most recent years of our study, along with varying patterns across age groups, regions, and cancer types, although significant joinpoints should not be taken to imply abrupt changes in underlying trends in risk.^19^ The variability we found warrants continued monitoring of incidence patterns in large populations over long periods, as trends that are not significant over short intervals might result in significant increases over a longer timeframe, and grouping smaller populations with no trend could yield a significant change in a pooled dataset. These considerations might also explain the discrepancy in the measured rate of change between our study and smaller European datasets.^3^, ^4^, ^5^, ^6^, ^7^, ^8^, ^9^, ^10^

Kroll and colleagues^24^ have linked the increase in childhood cancer incidence in Great Britain with advances in diagnostic technology and improved cancer registration, because of the concurrence of a step increase in leukaemia and non-CNS solid tumour incidence in 2002 with a registration plan enacted in 2001. Similarly, the rise in the incidence of CNS tumours in 1992 coincided with the introduction of innovative methods of diagnosis. As a result, childhood cancer might have been under-reported in Great Britain during parts of the period assessed in this study. Although we are not aware of similar studies in other countries, analogous effects on childhood cancer incidence cannot be excluded elsewhere in Europe and could have affected our results.

The gradual convergence of environmental factors, lifestyle, and health services across Europe might have contributed to the similarities in incidence trends across the regions, although such changes will probably have less of an effect on cancer in childhood than in older age. Nevertheless, some opposing trends (notably for lymphoma in the east) suggest caution should be taken in ascribing the slowly increasing trends exclusively to improved detection, at least before further detailed analyses by cancer type, age group, and geographical region are done.

Overall incidence in children is weighted towards leukaemia, which, in 75% of cases, is the precursor B-cell lymphoid leukaemia. The age-specific curve for this cancer type currently peaks around age 2–4 years in most high-income countries. With increasing social development over time, this peak shifts towards younger ages and becomes more pronounced.^2^, ^25^ This change is unlikely to be driven by improved registration, which would affect all ages in the same way. The deficit of lymphoid leukaemia cases seen in poorer settings can be ascribed in part to underdiagnosis,^26^, ^27^ but inequalities across social strata might also operate through relevant exposures, such as parental occupation, diet, or reproduction characteristics.^28^ Continuous socioeconomic development and increasing awareness of primary care practitioners might have contributed to the observed increase in leukaemia cases, although reasons for the stabilisation of leukaemia incidence in northern Europe are unclear.

Incidence of lymphoma was relatively high compared with incidence of leukaemia in children in eastern Europe. Considering the likely infectious origin of lymphoma at young ages,^29^ our observed decreasing incidence could be attributed to elimination of certain viral exposures. Furthermore, diagnosis of lymphoma might be delayed until older ages, especially if the decreasing trend in children is accompanied by an increasing trend in adolescents, as seen in the east over the second decade. Possible selective under-reporting could also be considered, as the incidence of leukaemia and other cancers continues to increase in children in the east. The high incidence in southern Europe is consistent with previous reports of possible environmental exposures.^30^ A further assessment of lymphoma trends by diagnostic subgroups, narrower age groups, and sex could help provide a more specific explanation of the observed temporal changes.

The stability of malignant CNS tumour incidence in adolescents is encouraging, although drawing firm conclusions should be postponed until incidence trends are examined using data that include non-malignant tumours in the populations in which these are ascertained completely to exclude the possible effect of changes in classification of tumours. In children, because of the shown persisting increase in incidence in malignant CNS tumours, and the exclusion of non-malignant tumours—specifically pilocytic astrocytoma—from our analyses, a further detailed assessment is warranted, especially considering the poor outcome for such tumours.

The trends for all other cancers combined show that the overall increase in incidence is not explained solely by changes in the three major groups of childhood and adolescent cancer. This fact is especially relevant in adolescents, as many cases are germ cell tumours and carcinomas.^1^ The candidate explanatory groups for the increase in at least a part of the period are thyroid carcinoma,^31^ testicular tumours,^32^ and melanoma.^33^ In particular, the patterns for Belarus are probably shaped by the pronounced increase and later waning of thyroid cancer incidence during the study period, reflecting exposure to the radioactive fallout from the Chernobyl accident.^34^ These observations also merit detailed studies.

In summary, in this study we aimed to provide an overview of cancer incidence trends in children and adolescents in Europe, as an introduction to a detailed investigation of patterns and trends and their possible determinants by diagnostic group and other subcategories in a concerted series of studies. We found a continued increase in cancer incidence in children and adolescents in Europe over the 20-year period 1991–2010. The diversity of trends across the regions and tumour groups warrants further monitoring and a more detailed assessment of the high-quality data collected by cancer registries, by specific tumour types and population subgroups. Whether the observed increase in cancer incidence in children and adolescents is due to enhanced discovery of cases or changing risk factors, the known frequency of cancer in young people should be considered in cancer control programmes. Without an accurate quantification of the possible causes of this increase, whether real or artifactual, it is prudent to continue aetiological research into the causes of cancer in children and adolescents and to explore possible preventive measures.
