# Supplementary material for: Changing geographical patterns and trends in cancer incidence in children and adolescents in Europe, 1991–2010 (Automated Childhood Cancer Information System): a population-based study
Source: Lancet Oncol. 2018 Sep;19(9):1159–69. doi: 10.1016/S1470-2045(18)30423-6 (PMC6120055; doi:10.1016/S1470-2045(18)30423-6)
Supplement: Supplementary appendix [file mmc1.pdf]

THE LANCET
Oncology

Supplementary appendix

This appendix formed part of the original submission and has been peer reviewed. We post it as supplied by the authors.

Supplement to: Steliarova-Foucher E, Fidler M M, Colombet M, et al. Changing geographical patterns and trends in cancer incidence in children and adolescents in Europe, 1991–2010 (Automated Childhood Cancer Information System): a population-based study. *Lancet Oncol* 2018; published online Aug 8. http://dx.doi.org/10.1016/S1470-2045(18)30423-6.

Changing geographical patterns and trends in cancer incidence in children and adolescents in Europe, 1991-2010 (Automated Childhood Cancer Information System): a population-based study

ACCIS contributors

Austria: M Hackl (Austrian National Cancer Registry); Belarus: A Zborovskaya (Childhood Cancer SubRegistry of Belarus); Bulgaria: N Dimitrova, Z Valerianova (Bulgarian National Cancer Registry); Czech Republic: M Dušek (Czech National Cancer Registry); Estonia: M Mägi (Estonian Cancer Registry); France: A Monnereau (French Cancer Incidence and Mortality Network (FRANCIM)); J Clavel (National Registry of Childhood Haematological Malignancies); M Velten (Bas-Rhin Cancer Registry); AV Guizard (Calvados General Cancer Registry); V Bouvier (Calvados Registry of Digestive Tumours); X Troussard (Lower Normandy Hemopathy Registry); AS Woronoff (Doubs Cancer Registry); E Marrer (Haut-Rhin Cancer Registry); B Tretarre (Hérault Cancer Registry); M Colonna (Isère Cancer Registry); O Ganry (Somme Cancer Registry); P Grosclaude (Tarn Cancer Registry); Germany: P Kaatsch (German Childhood Cancer Registry (GCCR)); B Holleczek (Saarland Cancer Registry); Hungary: Z Jakab (Hungarian Childhood Cancer Registry); Iceland: L Tryggvadóttir (Icelandic Cancer Registry); Italy: L Mangone (Italian Association of Cancer Registries (AIRTUM)); F Merletti (Childhood Cancer Registry of Piedmont); S Ferretti (Ferrara Province Cancer Registry); B Caruso (Tumour Registry of Modena); M Michiara (Parma Cancer Registry); R Tumino (Ragusa Cancer Registry); F Falcini (Romagna Cancer Registry); R Zanetti (Piedmont Cancer Registry, city of Turin); G Tagliabue (Lombardy Cancer Registry, Varese Province); Netherlands: O Visser (Netherlands Cancer Registry); Norway: G Ursin (Cancer Registry of Norway); Poland: R Mężyk (Kielce Cancer Registry); K Kepska (Lower Silesia Cancer Registry); Portugal: J Laranja Pontes (Portugal North Region Cancer Registry); Slovenia: M Primic Žakelj (Cancer Registry of Republic of Slovenia); Spain: R Fernández-Delgado (Spanish Registry of Childhood Tumours (RETI-SEHOP), UVEG); ML Vicente Raneda (Comunitat Valenciana Childhood Cancer Registry); E Almar Marqués (Albacete Cancer Registry, Castile-La Mancha); JR Quirós Garcia (Asturias Cancer Registry); A Lopez de Munain (Basque Country Cancer Registry); R Marcos-Gragera (Girona Cancer Registry); MJ Sanchez-Perez (Granada Cancer Registry, CIBERESP); M Ramos Monserrat (Mallorca Cancer Registry); E Ardanaz (Navarra Cancer Registry, CIBERESP); J Galceran (Tarragona Cancer Registry); Sweden: S Khan (Swedish Cancer Registry); Switzerland: CE Kuehni (Swiss Childhood Cancer Registry); C Bouchardy (Geneva Cancer Registry); F Levi (Neuchâtel Cancer Registry and Vaud Cancer Registry); I Konzelmann (Valais Cancer Registry); S Rohrmann (Cancer Registry Zurich and Zug); UK: CA Stiller (National Registry of Childhood Tumours); S Vernon (Public Health England); DH Brewster (Scottish Cancer Registry); C White (Welsh Cancer Registry); International Agency for Research on Cancer: M Colombet, A Dolya, E Steliarova-Foucher.

Changing geographical patterns and trends in cancer incidence in children and adolescents in Europe, 1991-2010 (Automated Childhood Cancer Information System): a population-based study

Table S-1. Overview of the datasets provided by the contributing population-based cancer registries.

Person-years and numbers of cases refer to age 0-19 (general cancer registries and Belarus) and age 0-14 (the other paediatric registries). DCO, death certificate only; HM, haematological malignancies; NOS, unspecified cases; MV, microscopic verification; Unkn., unknown. [1]Includes non-malignant tumours, pilocytic astrocytoma, myelodysplasia, skin cancer, and carcinoid of appendix.

Registry	Region	Contributing to analyses			Person-years	Total cases (A)	Excluded[1] (% A)	Included cases (B)	Basis of diagnosis (% B)			NOS (% B)
		0-14	0-14, HM	15-19					DCO	MV	Unkn.	
BELARUS, paediatric	East	+	+	+	51 608 997	8 477	5.2	8 035	0.1	97.2	0.0	4.1
BULGARIA	East	+	+	+	36 628 541	4 476	2.0	4 386	6.0	90.3	0.0	12.1
CZECH REPUBLIC	East	+	+	+	49 098 564	8 306	6.9	7 732	1.6	92.9	0.0	7.7
HUNGARY, paediatric	East	+	+		34 416 963	4 756	8.2	4 368	—	97.5	0.0	0.7
POLAND, Kielce	East	+	+	+	6 570 051	909	1.4	896	0.0	91.7	0.6	13.1
POLAND, Lower Silesia	East	+	+	+	14 973 285	1 971	3.7	1 898	0.0	88.1	0.0	16.5
ESTONIA	North	+	+	+	7 026 036	1 103	9.2	1 001	0.5	96.7	0.0	4.6
ICELAND	North	+	+	+	1 744 021	314	11.1	279	0.0	94.6	0.0	6.5
NORWAY	North	+	+	+	23 361 679	4 185	15.5	3 537	0.1	96.5	0.0	3.2
SWEDEN	North	+	+	+	43 152 649	6 899	9.4	6 250	—	97.5	0.0	5.8
UK, England and Wales, paediatric	North	+	+		196 350 418	27 799	11.7	24 543	0.3	92.3	1.8	1.7
UK, England	North			+	247 079 350	34 873	5.4	33 004	0.3	91.2	2.7	4.2
UK, Scotland	North	+	+	+	24 640 383	3 890	12.3	3 411	0.2	95.6	1.6	2.8
UK, Wales	North			+	14 634 780	2 263	11.0	2 014	0.8	76.0	18.1	6.3
ITALY, Piedmont, paediatric	South	+	+		10 619 168	1 997	12.8	1 741	0.1	97.0	0.0	1.8
ITALY, Ferrara	South	+	+	+	982 434	231	8.2	212	0.9	85.4	0.0	12.7
ITALY, Modena	South	+	+	+	2 211 491	436	6.2	409	0.5	96.3	0.0	2.4
ITALY, Parma	South	+	+	+	1 292 841	286	8.7	261	0.0	96.9	0.0	6.1
ITALY, Ragusa	South	+	+	+	1 672 408	332	12.7	290	0.0	93.8	0.0	7.9
ITALY, Romagna	South	+	+	+	3 421 112	697	12.9	607	0.3	91.4	0.0	8.7
ITALY, Turin	South			+	2 850 497	511	0.4	509	0.2	96.1	0.4	4.9
ITALY, Varese	South	+	+	+	3 098 091	629	6.4	589	0.2	96.8	0.0	1.7
PORTUGAL, North	South	+	+	+	16 009 471	2 575	8.4	2 358	—	98.0	0.5	4.2
SLOVENIA	South	+	+	+	9 125 219	1 411	7.5	1 305	0.0	99.2	0.0	2.0
SPAIN, Selected regions, paediatric	South	+	+		39 566 669	5 892	6.9	5 487	—	92.9	0.1	1.8
SPAIN, Valencia, paediatric	South	+	+		13 540 211	2 204	7.2	2 046	0.0	94.9	0.0	1.8
SPAIN, Albacete	South	+	+	+	1 752 522	286	5.9	269	0.4	96.3	0.0	3.0
SPAIN, Asturias	South	+	+	+	3 708 214	599	4.8	570	0.2	96.7	0.0	4.4
SPAIN, Basque Country	South			+	8 073 240	1 520	8.6	1 390	0.6	95.8	0.0	6.4
SPAIN, Girona	South			+	2 567 924	475	9.9	428	0.5	97.0	0.5	1.9
SPAIN, Granada	South	+	+	+	4 262 321	689	8.6	630	0.3	95.9	0.0	3.7
SPAIN, Mallorca	South	+	+	+	3 142 488	533	7.9	491	0.0	95.7	0.8	2.6
SPAIN, Navarra	South			+	2 308 507	459	8.9	418	1.0	95.9	0.0	2.9
SPAIN, Tarragona	South			+	2 779 386	517	6.6	483	0.4	96.3	0.0	2.3
AUSTRIA	West	+	+	+	36 609 850	5 886	5.0	5 593	1.6	97.4	0.4	5.1
FRANCE, paediatric, HM	West		+		225 636 031	13 306	1.0	13 178	—	97.9	0.0	1.3
FRANCE, Bas-Rhin	West	+		+	5 290 643	909	8.8	829	—	95.8	0.0	3.1
FRANCE, Calvados	West	+		+	3 490 024	556	7.9	512	—	97.9	0.0	3.3
FRANCE, Doubs	West	+		+	2 699 695	451	7.8	416	—	97.6	0.0	3.6
FRANCE, Haut-Rhin	West	+		+	3 696 586	603	7.8	556	—	98.6	0.0	3.2
FRANCE, Hérault	West	+		+	4 500 673	785	6.8	732	—	96.9	0.0	1.4
FRANCE, Isère	West	+		+	6 040 465	1 074	8.6	982	—	96.9	0.1	1.6
FRANCE, Somme	West	+		+	2 971 919	416	2.2	407	—	95.6	0.0	2.0
FRANCE, Tarn	West	+		+	1 613 625	256	11.7	226	—	97.3	0.0	2.2
GERMANY, paediatric	West	+	+		248 679 583	36 006	10.2	32 324	—	96.6	0.0	0.8
GERMANY, Saarland	West			+	4 145 262	714	9.7	645	1.1	95.7	0.0	12.1
THE NETHERLANDS	West	+	+	+	77 479 099	12 797	10.7	11 423	—	97.1	0.0	1.9
SWITZERLAND, paediatric	West	+	+		24 300 913	3 696	10.5	3 309	0.2	93.5	0.9	1.5
SWITZERLAND, Geneva	West			+	1 817 468	305	11.1	271	0.0	96.7	0.0	1.1
SWITZERLAND, Neuchâtel	West			+	761 176	152	9.2	138	0.7	98.6	0.0	1.4
SWITZERLAND, Valais	West			+	1 332 868	214	11.7	189	0.5	97.9	0.0	2.6
SWITZERLAND, Vaud	West			+	2 910 584	489	9.4	443	0.7	98.4	0.0	2.9
SWITZERLAND, Zurich	West			+	5 074 230	972	10.1	874	0.2	98.2	0.0	3.5

Changing geographical patterns and trends in cancer incidence in children and adolescents in Europe, 1991-2010 (Automated Childhood Cancer Information System): a population-based study

Table S-2. Overview of the data available for analyses. Europe, 1991-2010.

CNS, central nervous system; DCO, death certificate only; MV, microscopically verified; N_0, total number available in the selected registries over the defined study period; N number of cases included in various analyses; NOS, unspecified histology; PA, pilocytic astrocytoma of any behaviour; Unkn., unknown.
[1]UN population estimates for Europe and the regions in 2010 [Reference], the year used for calculation of coverage.
[2]The percentage was calculated by comparing the average annual population in the areas covered by the participating registries (numerator) and the UN estimates of the total population in the four European regions and the two age groups (denominator).
[3]The percentage relates to the total numbers of cases, N_0.
[4]The percentage relates to the included numbers of cases, N.
[5]Only the areas with access to death certificates were included in the calculation of the percentage.
[6]Population data for 2010 (the denominator) include Cyprus
[7]The person-years shown are those included in the analyses of haematopoietic malignancies; the person-years included in the analyses of all cancers and the CNS tumours was 380 456 382. N includes all 13 178 haematopoietic malignancies (HM) from the French national paediatric registry and the 1723 cases classified into other diagnostic groups from the French general cancer registries, but excludes 1408 HM registered in the general cancer registries.

Age and Region	Year 2010		Person-years included	Cases								Basis of diagnosis[4]			
	Population[1] (millions)	Coverage[2] %		Total	Excluded[3]					Included		MV	DCO[5]	Unkn.	NOS
				N_0	%	Non-malignant CNS tumours and PA	Myelo-dysplasias	Skin cancer	Carcinoid of appendix	N		%	%	%	%
**Age 0-14 years**															
**East**	43·7	13·7	145 896 608	20 483	5·9	1 019	65	105	20	19 274		93·9	1·9	0·0	6·8
**North**	17·5	76·9	270 670 172	38 954	11·8	4 130	227	225	33	34 339		93·4	0·3	1·4	2·5
**South**[6]	23·0	25·5	99 832 251	15 745	8·2	1 203	32	47	13	14 450		94·7	0·1	0·1	2·7
**West**[7]	29·9	93·3	583 782 306	67 516	8·2	4 720	642	50	132	61 972		96·6	1·0	0·1	1·4
**Europe**[6]	114·1	46·6	1 100 181 337	142 698	8·9	11 072	966	427	198	130 035		95·2	0·8	0·4	2·6
**Age 15-19 years**															
**East**	18·2	10·2	47 399 793	8 412	4·4	134	26	180	31	8 041		94·6	1·5	0·0	7·6
**North**	6·4	76·9	90 971 926	16 231	6·3	747	12	178	87	15 207		93·1	0·4	2·5	5·4
**South**[6]	8·0	10·7	20 009 028	4 271	7·7	212	9	102	7	3 941		98·0	0·3	0·2	3·8
**West**[7]	10·6	20·1	41 048 267	8 723	8·9	474	40	41	219	7 949		98·7	1·1	0·1	3·0
**Europe**[6]	43·2	22·6	199 429 014	37 637	6·6	1 567	87	501	344	35 138		95·3	0·8	1·1	5·2

Reference:

UN Department of Economic and Social Affairs, Population Division. World Population Prospects 2017. https://esa.un.org/unpd/wpp/ (accessed Feb 21, 2018)

Figure S-1. Incidence trends of leukaemia, lymphoma, malignant CNS tumours and other cancers in children aged 0-14 years, by region. Europe, 1991-2010.

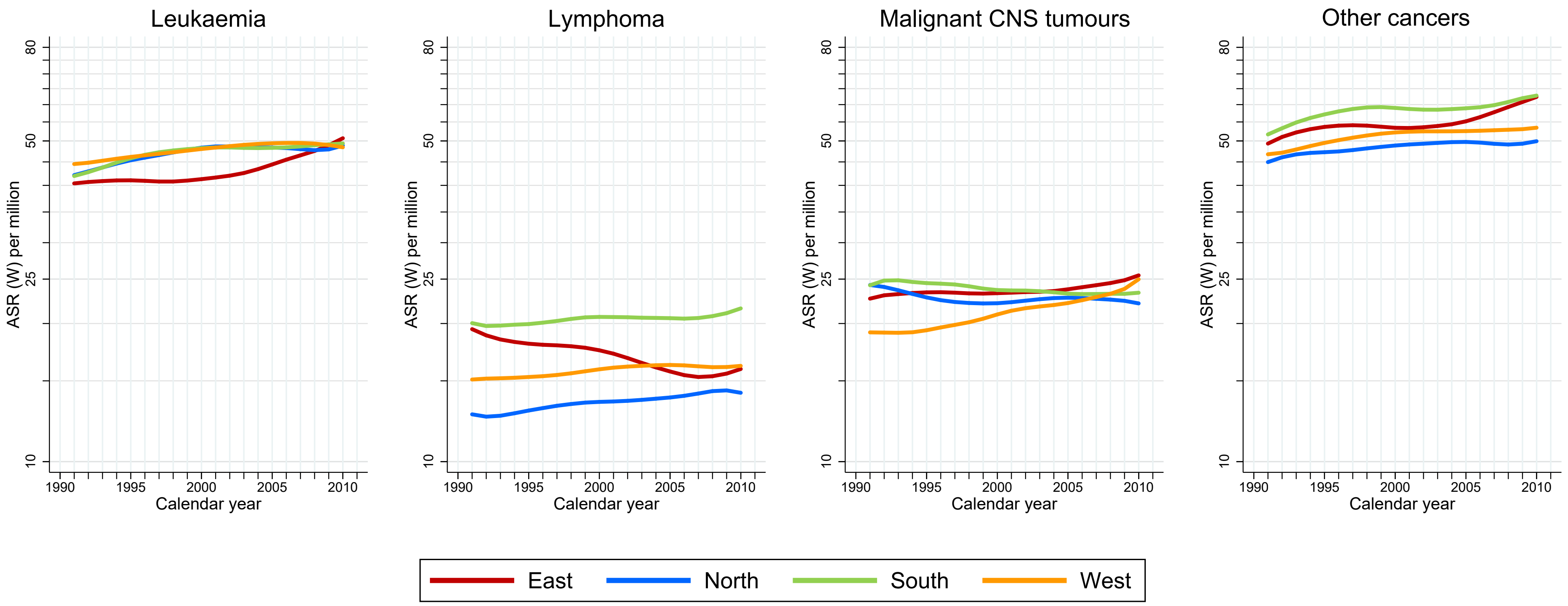

Figure S-2. Incidence trends of leukaemia, lymphoma, malignant CNS tumours and other cancers in adolescents aged 15-19 years, by region. Europe, 1991-2010.

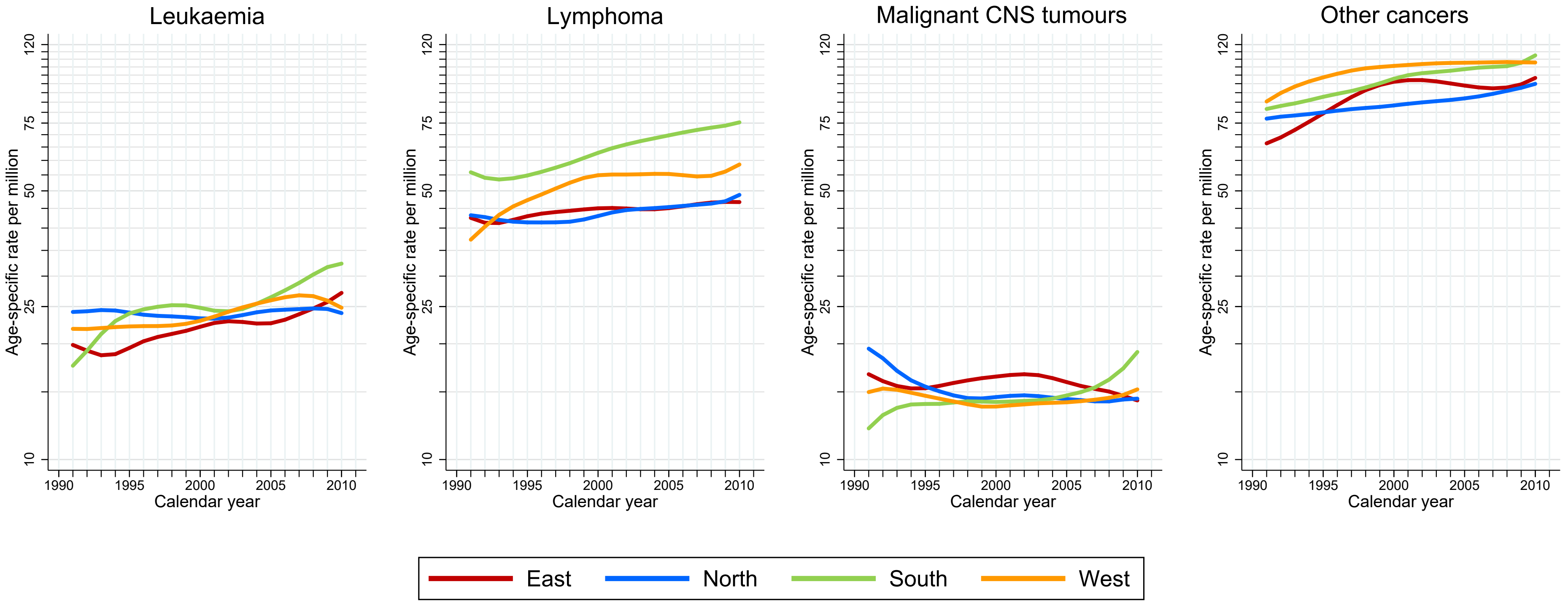

Figure S-3. Schematic presentation of changes in cancer incidence trends in children (age 0-14 years) and adolescents (age 15-19 years) by region of Europe for the total period 1991-2010 (Overall) and for the constituent time segments with significantly different trends if detected in the joinpoint analysis (Segments).

↑significant increase; ↓significant decrease; → stable (non-significant) trend. Where no segments are shown, no joinpoints were detected.

	All cancers		Leukaemia		Lymphoma		Malignant CNS tumours		Other cancers	
	Overall	Segments	Overall	Segments	Overall	Segments	Overall	Segments	Overall	Segments
**Age 0-14 years**										
**East**	↑	→→	↑		↓		→		↑	
**North**	↑		↑	↑→	→		→		→	
**South**	↑		↑		→		→		↑	
**West**	↑		↑		↑		↑		↑	↑→
**Europe**	↑		↑		→		↑		↑	↑→→
**Age 15-19 years**										
**East**	↑	→↑↓→	↑		↑	→→→↑	→		↑	↑↓→
**North**	↑	→→↑	→		↑		→		↑	
**South**	↑		↑		↑		→		↑	
**West**	↑	↑↑	↑		↑	↑→→	→		↑	↑→
**Europe**	↑	↑→→	↑		↑		→		↑	↑→→
